# Top IHC/ISH Hacks for and Molecular Surrogates of Poorly Differentiated Sinonasal Small Round Cell Tumors

**DOI:** 10.1007/s12105-023-01608-z

**Published:** 2024-02-05

**Authors:** Diana Bell

**Affiliations:** Anatomic Pathology, Disease Team Alignment: Head and Neck, City of Hope Comprehensive Cancer Center, 1500 E Duarte Rd, Duarte, CA 91010 USA

**Keywords:** Small round cell tumors, Sinonasal, Epithelial, Neuroectodermal, Immunohistochemistry, Molecular testing

## Abstract

**Background:**

Poorly differentiated sinonasal small round cell tumors (SRCTs) are rare and heterogeneous, posing challenges in diagnosis and treatment.

**Methods:**

Recent advances in molecular findings and diagnostic refinement have promoted better understanding and management of these tumors.

**Results:**

The newly defined and emerging sinonasal entities demonstrate diverse morphologies, specific genomic signatures, and clinical behavior from conventional counterparts. In this review of SRCTs, emphasis is placed on the diagnostic approach with the employment of a pertinent panel of immunohistochemistry studies and/or molecular tests, fine-tuned to the latest WHO 5 classification of sinonasal/paranasal tumors and personalized treatment.

**Conclusion:**

Specifically, this review focuses on tumors with epithelial and neuroectodermal derivation.

**Supplementary Information:**

The online version contains supplementary material available at 10.1007/s12105-023-01608-z.

## Introduction

Variegated epithelial, neuroectodermal, mesenchymal, and hematolymphoid neoplasms arise in the sinonasal cavities, accounting for approximately 3–5% of all head and neck tumors. With a combined incidence of 0.5–1.0 cases per 100,000 per year, sinonasal malignancies are considered rare cancers. While rare tumors account for approximately 20% of all cancer patients, new advances lag behind those reported in more common solid tumors, with few clinical trials currently benefiting these patients [1, 2]. Moreover, sinonasal tumors present challenges to clinical management due to anatomic considerations, along with their distinctive etiologies, epidemiology, clinical and genetic characteristics. Nevertheless, progress has been made in recent decades regarding surgical techniques, imaging modalities, and radiotherapy and in the identification of molecular alterations that may improve diagnosis, identification of new entities, prognosis, and the stratification of treatment.

Sinonasal small round cell tumors (SRCTs) constitute a heterogeneous group of malignant neoplasms characterized by a monotonous population of undifferentiated tumor cells with a relatively high nuclear to cytoplasmic ratio and high mitotic activity in conventional H&E light microscopy. An early and accurate diagnosis is imperative so patients with paranasal/nasal cavity SRBCTs can undergo appropriate therapy. Completing a definitive diagnosis of an SRCT based solely on H&E findings may be exceedingly difficult because of the frequent absence of distinguishing features. In routine practice, additional challenges include suboptimal diagnostic tissue (small, crushed, poorly preserved, necrotic, fibrotic, predominantly blood clot) and sampling errors [3]. For an accurate diagnosis in this group of tumors, there is a heavy reliance on ancillary studies, including a broad panel of immunohistochemical stains and molecular studies [3].

In this review of SRCTs, emphasis is placed on the diagnostic approach with the employment of a pertinent panel of immunohistochemistry studies and/or molecular tests fine-tuned to the latest WHO 5 classification of sinonasal/paranasal tumors and personalized treatment.

## Poorly Differentiated High-Grade Sinonasal Carcinomas

### Nuclear Protein in Testis (NUT) Carcinoma (NC)

Nuclear protein in testis (NUT) carcinomas (NCs) are rare, clinically aggressive carcinomas that are characterized by a translocation involving the *NUTM1* gene on chromosome 15q14 and, in most cases (~ 70–80%), the bromodomain-containing 4 (*BRD4*) gene on chromosome 19p13.1, resulting in a *BRD4-NUTM1* fusion oncogene [4, 5]. Other variant rearrangements include the *BRD3-NUTM1* fusion (~ 15–20%) [6] and *NSD3-NUTM1* fusion (~ 6%) [7], among partner genes (zinc finger ZNF52, ZNF592 in ~ 2%) [8]. In a subset of malignant solid tumors from soft tissue and other organs of uncertain relationship to NCs, *NUTM1* has been reported to be fused with other genes (*YAP1*, *MXD1*, *MXD4*, *CIC*, *BCORL1*, *ATXN1*, and *MGA)*; these genes have been described to occur in high-grade sarcoma associated with a distinct pathogenetic pathway (reviewed in Moreno et al. [9]).

NCs are composed of undifferentiated basaloid cells with focal, often abrupt, squamous differentiation [10]. NCs can mimic other undifferentiated neoplasms, such as pediatric small blue cell tumors, germ cell tumors, Ewing sarcoma, lymphoma, or SNUC. NUT carcinomas have an epithelial immunophenotype and focally express keratin, p63, CK7, CK20, and CK34, which reflect varying degrees of squamous differentiation. An extensive panel of lineage immunomarkers (*e.g.,* desmin, myoglobin, smooth muscle actin, muscle actin, chromogranin, synaptophysin, leukocyte common antigen, placental alkaline phosphatase, S100 protein, alpha fetoprotein, neuron-specific enolase, CD57, CD99, HMB45) are not expressed in NCs. Oncoviruses, such as Epstein‒Barr virus and HPV, have not been reported thus far in NCs; their presence would likely exclude this diagnosis. Demonstration of the NUT translocation is needed for definitive diagnosis of NCs; this can be achieved by karyotyping, reverse transcription polymerase chain reaction, fluorescence in situ hybridization (FISH), and next-generation sequencing (NGS)- or whole-exome sequencing (WES)-based approaches (reviewed in Moreno et al. [9]).

Immunohistochemistry for NUT represents an acceptable surrogate marker, with NCs showing a nuclear staining pattern. Immunostaining with a monoclonal antibody to NUT has a sensitivity of 87%, a specificity of 100%, a negative predictive value of 99%, and a positive predictive value of 100% in distinguishing NCs from other poorly differentiated sinonasal carcinomas [11]. Given the anecdotal favorable responses of NUTs to certain treatment regimens, including chemotherapy according to Ewing sarcoma protocols or docetaxel and radiotherapy [12, 13], the distinction of NCs from other sinonasal carcinomas appears to be of clinical relevance. Targeted therapy using small-molecule BET inhibitors has shown activity but no obvious survival benefits, most likely due to toxicity effects [14]. Any poorly differentiated midline carcinoma or head and neck tumor lacking lineage-specific differentiation markers should be considered for immunostaining for NUT or rearrangement testing.

*MYC* has been shown to be a downstream oncogene target of *BRD4::NUTM1* that blocks NC cellular differentiation and maintains a proliferative state [15]. The transcription factor SOX2 (sex-determining region Y-box protein 2), which is essential for stem cell self-renewal and pluripotency, is also an oncogenic target of *BRD4::NUTM1* [16, 17]. *BRD4::NUTM1* has been shown to drive overexpression of SOX2 in NUT carcinoma cells, which induces an aberrant stem cell-like growth feature [17]. Sox2 expression is normally restricted to stem cells; its aberrant overexpression has been linked to the ability to promote tumorigenicity and a poorly differentiated morphology [18–20]. Sox2 expression and gene amplification have been identified as common events in the head and neck [21, 22]; in the sinonasal region, amplification and/or overexpression of Sox2 has been demonstrated in squamous carcinoma (SNSCC), sinonasal undifferentiated carcinoma (SNUC), adenoid cystic carcinoma (AdCC), and intestinal type adenocarcinoma (ITAC) [22–24]. Although the literature is controversial regarding SOX2 amplification/Sox2 expression, recent data highlight the driver role of SOX2 in stemness with Sox2 overexpression and poor outcomes in patients with solid tumors [25]. Sox2 expression is also associated with resistance to chemotherapy through a plethora of mechanisms, and as such is a promising target for anticancer therapy [22, 26].

Tumor-specific antigens (TSAs) and tumor-associated antigens (TAAs) have been discovered within recent decades [27]. TSAs may result from gene mutations or from the expression of alternative open reading frames, resulting from chromosomal rearrangements; normal tissues frequently carry TAAs, with the drawback of autoimmunity development in parallel to conferring tolerance to these antigens through vaccination and tumor recognition [27–29]. **Pr**eferentially expressed **a**ntigen in **me**lanoma (PRAME) is a testis-selective cancer testis antigen with restricted expression in somatic tissues and re-expression in various cancers. PRAME has gained interest as a candidate target for immunotherapy [30]. PRAME plays a role in the acquisition of various cancer hallmarks, including replicative immortality or stemness, invasion, and metastasis [30]. In addition to supporting tumor features, PRAME has been implicated in the regulation of the immune response [31].

In a recent study that aimed to characterize the immune-oncology gene expression profile in sinonasal undifferentiated carcinomas (SNUCs) and other high-grade sinonasal carcinomas, PRAME was the top upregulated gene in SNUCs and SWI/SNF-deficient sinonasal carcinomas (fold change 8.40), and fold change half values (4.8) were observed for high-grade neuroendocrine carcinomas (HGNECs) [32]. PRAME protein overexpression has also been noted in some NUT carcinomas (D Bell unpublished observations).

**Fig. 1:**
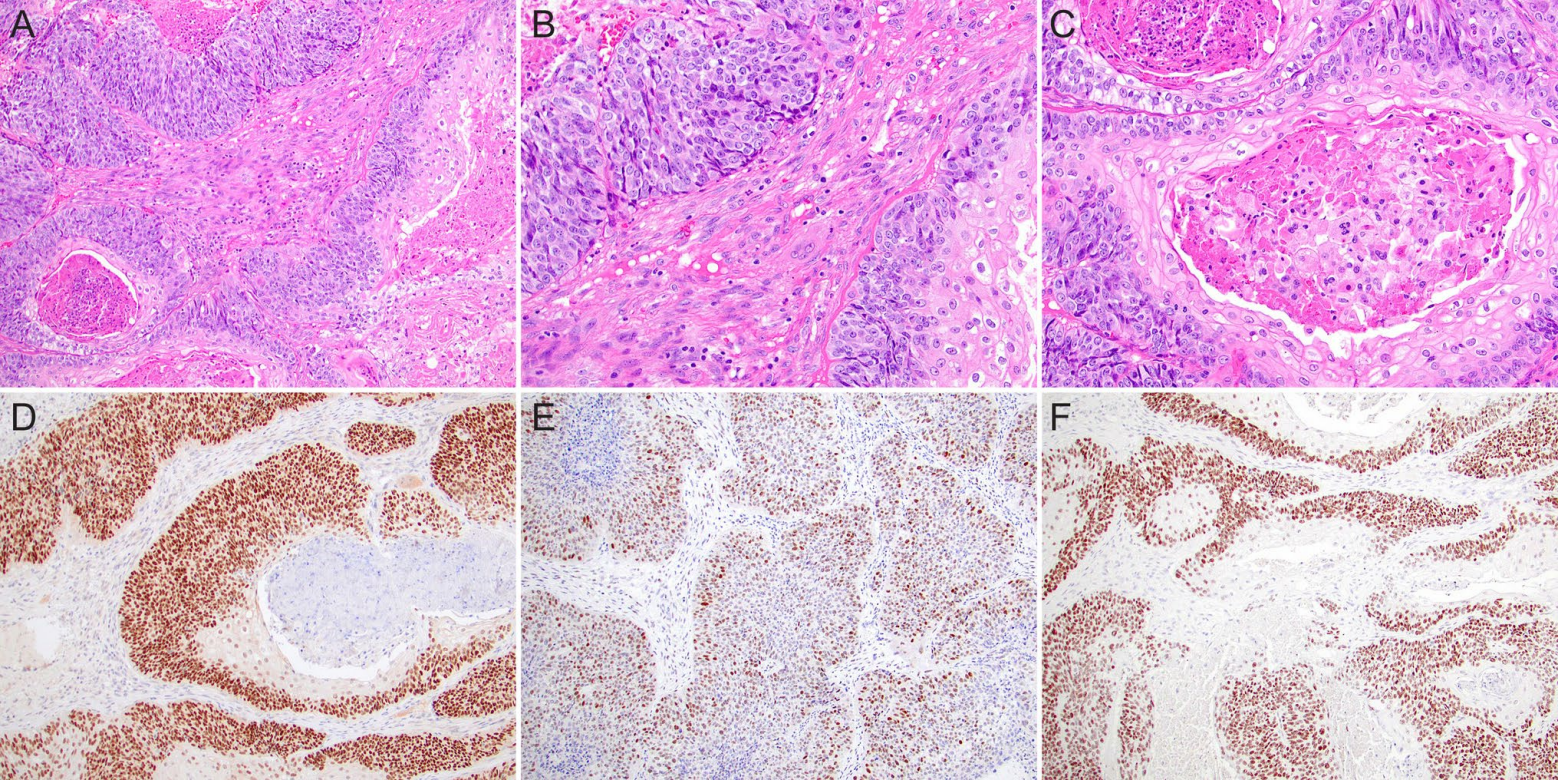
NUT carcinoma. (**A**, **B**) Salient morphological features-monotonous proliferation of round small-to-medium-sized cells; abrupt keratinization and foci of squamous differentiation with larger cells with eosinophilic cytoplasm and pearl formation (**C**). Immunoperoxidase staining with (**D**) anti-NUT, (**E**) anti-SOX2, (**F**) anti-PRAME

Salient morphological features of NUT carcinoma, with immunohistochemistry for NUT surrogate diagnosis, SOX2 and PRAME as promising anticancer targets, are illustrated in Fig. 1.

### Sinonasal Lymphoepithelial Carcinoma (SLEC)

Similar to other anatomical sites, sinonasal lymphoepithelial carcinoma (SLEC) is composed of sheets of undifferentiated malignant epithelial cells intimately intermingled with chronic inflammatory infiltrate. Malignant cells are often EBV positive [33], as shown by in situ hybridization for EBV-encoded RNA (EBER); serology for EBV-encoded RNA is also available.

The presence of lymphocytic infiltration, EBV expression, and the lack of neuroendocrine markers helps to differentiate SLEC mainly from SNUC and HGNEC.

High expression of somatostatin receptor 2 (SSTR2) has been documented in nasopharyngeal lymphoepithelial carcinoma, thymic LEC and salivary LEC [34–38]. SSTR2, a G-protein-coupled cell surface receptor, inhibits cell proliferation and is mainly expressed in neuroendocrine tumors. Lechner et al., in their large cohort of nasopharyngeal carcinomas, proposed a prognostic role for SSTR2 expression, with higher expression associated with increased survival rates [35]. High expression of SSTR2 is helpful as a diagnostic biomarker by imaging and an increased uptake of specific radiocontrast in EBV + NPC [39]. Targeted therapeutic strategies with SSTR2 agonists have also been studied with agonists prolonging progression-free survival in patients with metastatic enteropancreatic neuroendocrine tumors grade 1 or 2 (Ki67 < 10%) [40].

**Fig. 2:**
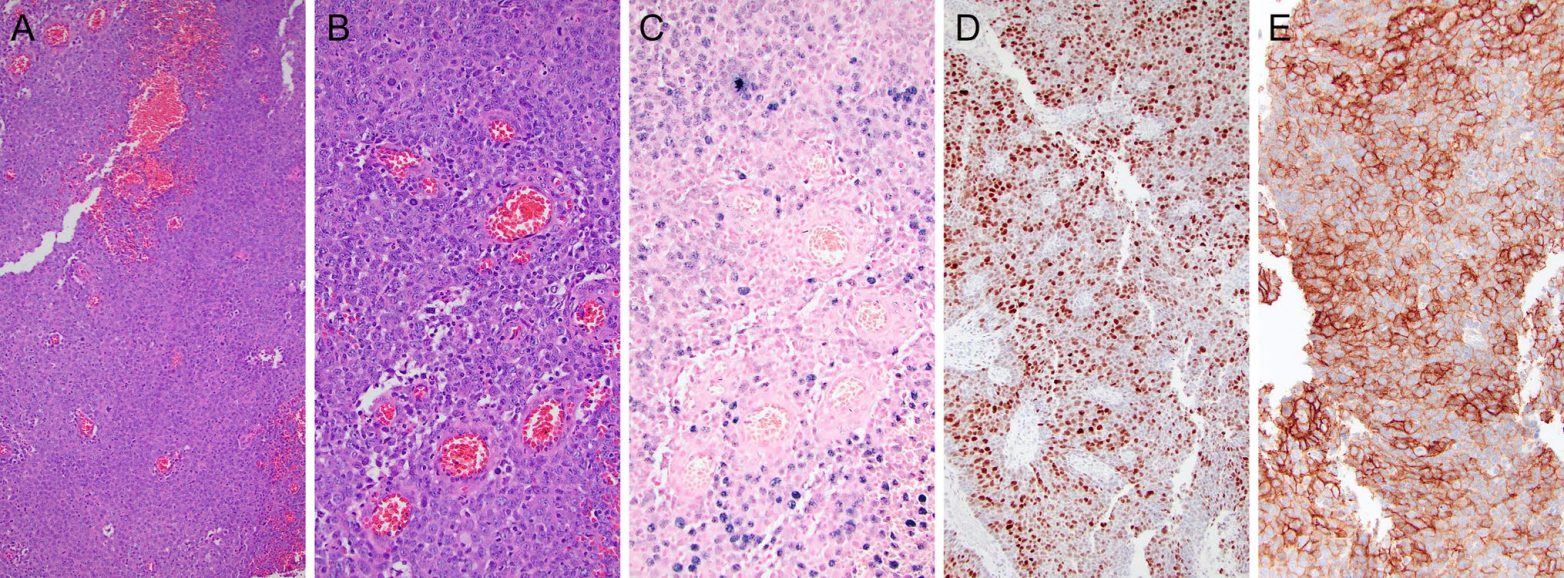
Sinonasal lymphoepithelial carcinoma. (**A**, **B**) H&E, (**C**) Presence of Epstein-Barr virus confirmed by EBER in situ hybridization. Immunostaining with (**D**) PRAME and (**E**) SSTR2

In view of SSTR2 diagnostic, imaging and therapeutic implications extrapolated from nasopharyngeal carcinoma studies, along with SSTR2 sensitivity and specificity for LEC, SSTR2 testing in SLECs is encouraged. Figure 2 depicts a nasal septum EBER-positive undifferentiated carcinoma, with strong expression of PRAME and SSTR2.

### Sinonasal Undifferentiated Carcinoma (SNUC) and IDH2-Mutant Sinonasal Carcinomas

The WHO redefined SNUC as a highly aggressive and clinicopathologically distinct carcinoma of uncertain histogenesis that typically presents with great local aggressivity and tendency to metastasize [41]. SNUC is reputed to be refractory to even the most radical therapy and to carry a poor prognosis, particularly when the tumor transgresses the cranial base [42, 43].

In general, SNUC presents high chemosensitivity to cisplatin-based regimens, and a partial or complete response to induction chemotherapy is considered a favorable prognostic factor. Definitive chemoradiation is therefore usually recommended, while surgery is used as a salvage treatment in cases of persistence or recurrence [44].

SNUC arises from the sinonasal epithelium and therefore is of ectodermal derivation. In light of the overlapping clinical, anatomical, microscopic, and ultrastructural findings in olfactory neuroblastoma (ONB) and neuroendocrine carcinoma (NEC), their origins may share both cells of sinonasal respiratory mucosa and cells of olfactory neuroepithelium [45]. It has also been proposed that SNUC would be best categorized as a large-cell neuroendocrine carcinoma (reviewed in [46]).

SNUC is regarded as a diagnosis of exclusion. The immunohistochemical panel stains positively for epithelial markers (AE1/AE3, CK7, CAM5.2, EMA), p16, CD117, and focal p63 and negatively for CK5/6, p40, CEA, EBER, CD34, desmin, S100 protein, and calretinin. Neuroendocrine markers (synaptophysin, chromogranin, INSM, CD56) may be present.

An SNUC subtype with mutations in the Krebs cycle enzyme IDH2 is well characterized. IDH2 p.R172S is the most common mutation (55%); other mutations in the same codon (R172M, R172T, and R172G) have been described, and IDH1 mutations have rarely been reported [41, 47]. The spectrum is expanding, with IDH2 mutations documented in poorly differentiated high-grade carcinomas occurring in the sinonasal/paranasal anatomical boundaries as well as a handful of high-grade olfactory neuroblastomas [48]. As hypermethylation and upregulation of the repressive H3K27 epigenetic mark are hallmarks of IDH2-mutated carcinomas, DNA methylation-based classification is conceivable [48]. Given the therapeutic implications of IDH inhibitors, paralleling acute myeloid leukemia, some authors advocate for the classification of IDH2-mutated sinonasal tumors as a separate entity.

**Fig. 3:**
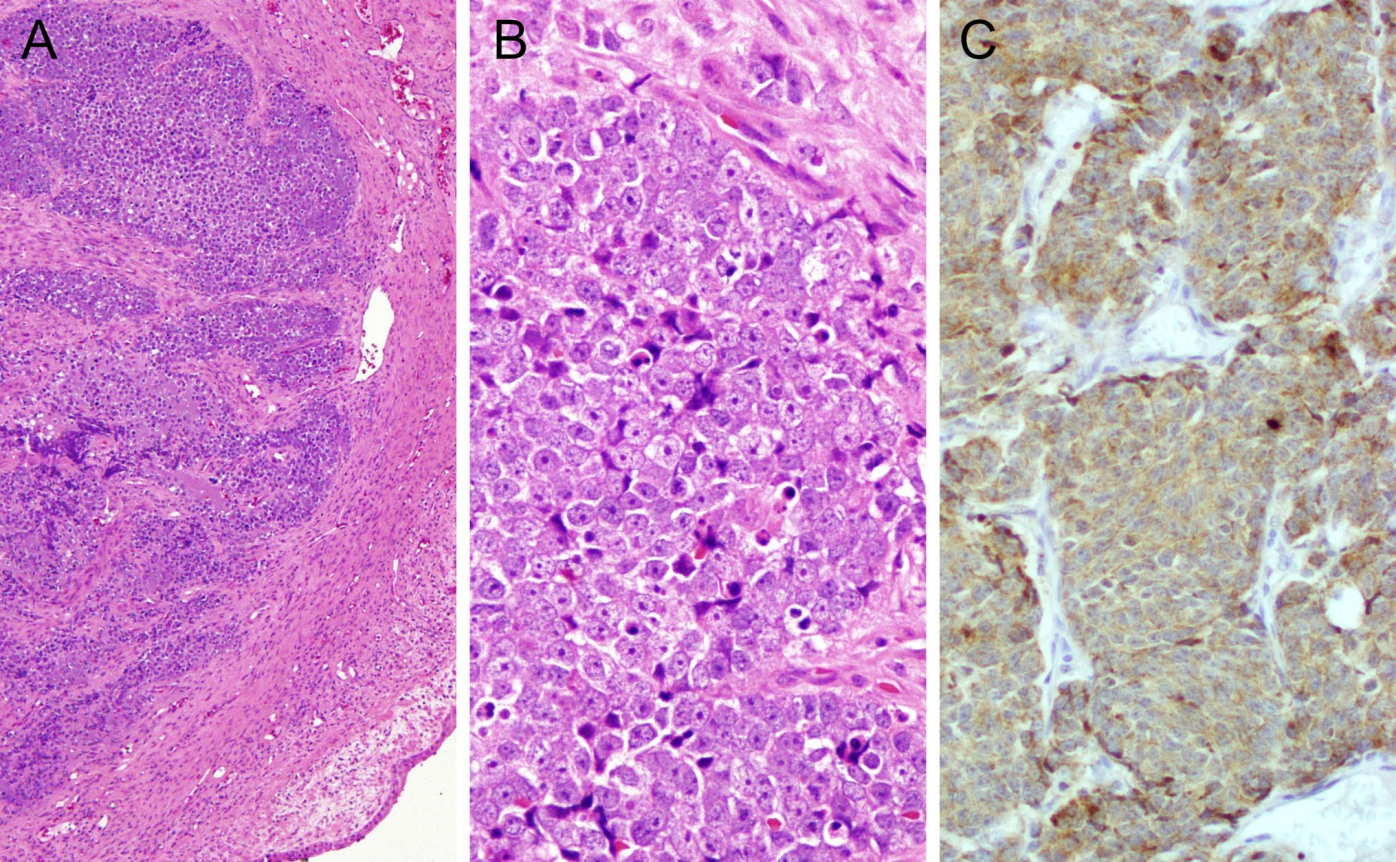
Sinonasal IDH2-mutated carcinoma. (**A**) H&E- Submucosal lobules of undifferentiated malignant cells, with large nuclei and prominent nucleoli (**B**) H&E. Diffuse immunoreactivity with anti-mutant IDH1/2 pR132/172 (**C**)

To date, no morphological or phenotypical differences between IDH-mutant and IDH-WT carcinomas have been recognized. Antibodies that recognize IDH1/2 (pR132/172) are a surrogate for diagnosis confirmation (granular cytoplasmic staining pattern); however, molecular testing validation is recommended. An example of a maxillary SNUC IDH2 mutation is shown in Fig. 3; the genomic event was confirmed by NGS studies.

### SWI/SNF Complex-Deficient Sinonasal Carcinomas

Sinonasal carcinomas characterized by rhabdoid/basaloid morphology and loss of expression of the SWI/SNF complex (SMARCB1, SMARCA4, SMARCA2), previously viewed as a subset of SNUCs, are recognized as a standalone entity in the 5th edition WHO Classification of Head and Neck Tumours [49]. Separation from the other types of sinonasal malignancies is justified, as the identification of SWI/SNF complex deficiency may provide a new target for novel treatment approaches and may ultimately lead to improved patient survival [50].

**Fig. 4:**
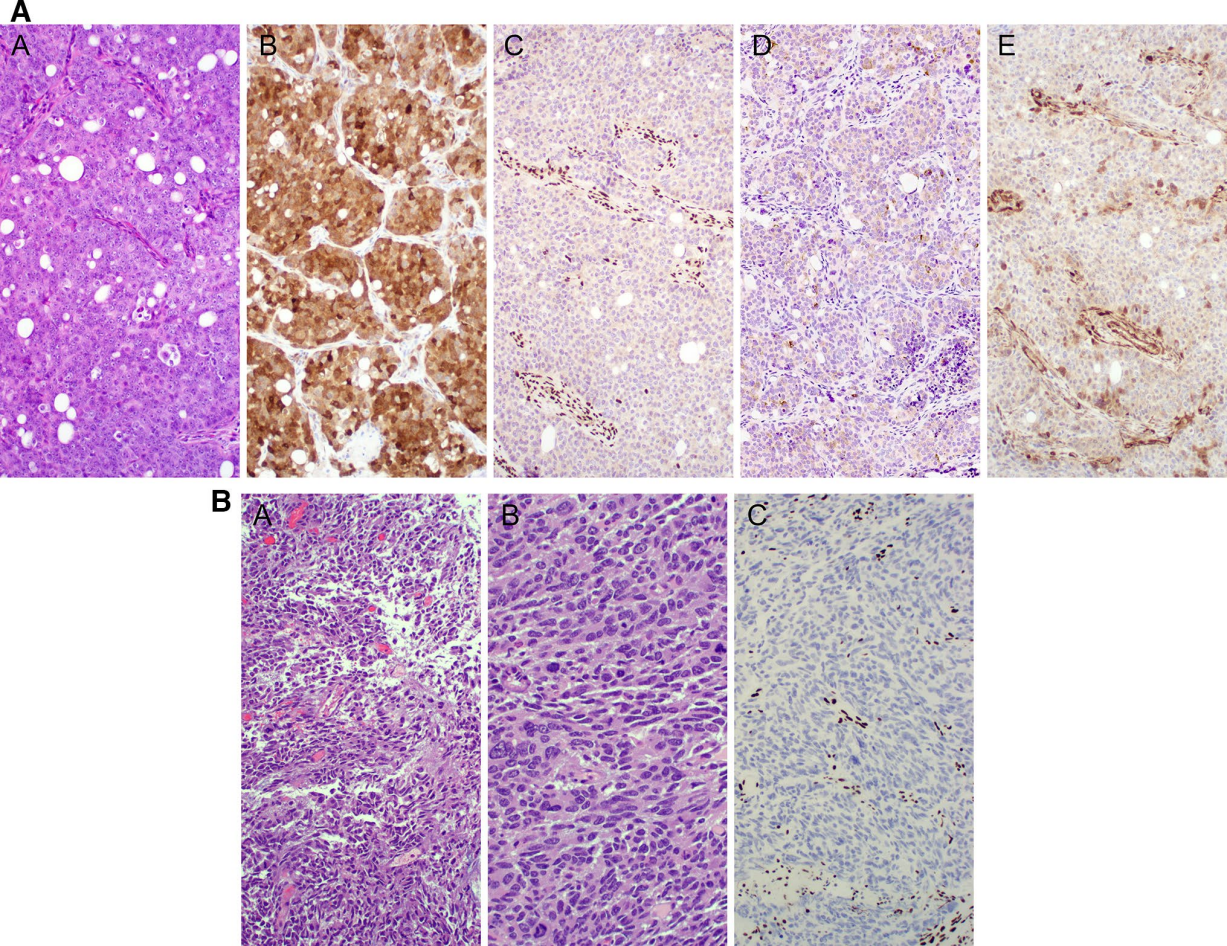
SNI/SWI complex-deficient sinonasal carcinoma. (**a**) SMARCB1/INI1-deficient sinonasal carcinoma. (**A**). H&E. Undifferentiated carcinoma (with large nuclei and prominent nucleoli) (**B**) Diffuse immunoreactivity with anti-p16. (**C**) Complete loss of expression of SMARCB1/INI1 (immunoperoxidase study with anti INI1/BAF47, with internal positive control/endothelial cells). (**D**) Patchy and weak expression of synaptophysin. E) Diffuse loss of expression of PTEN (correlated with NGS findings). (**b**) SMARCA4-deficient sinonasal carcinoma. (**A**, **B**) H&E- High-grade rhabdoid cells and rhabdoid appearance. (**C**) Complete loss of expression of SMARCA4 visualized by immunostaining with anti-BRG1 (vascular internal control highlights nuclear signal and retention)

Available antibodies for SMARCB1/INI1 (BAF47) and SMARCA4 (BRG1) are routinely employed surrogates (FISH and NGS molecular studies offer verification of these genomic alterations). The tumor is positive for pancytokeratin and variably positive for CK5/6, p63/p40, and CK7; focal reactivity for synaptophysin and chromogranin is evident. P16 immunostaining is often positive but is not associated with the presence of HPV. HPV, EBV, and NUT are negative. Complete loss of SMARCB1 (INI1) is mandatory (SMARCA4 expression is retained); conversely, loss of SMARCA4 (with preservation of SMARCB-1/INI1) is diagnostic (Fig. 4a, b). Co-loss of SMARCA2 is occasionally observed [51].

The spectrum of SWI/SNF-deficient sinonasal carcinomas currently includes the following: (1) SMARCB1-deficient sinonasal carcinoma, (2) SMARCB1-deficient sinonasal adenocarcinoma (with unequivocal glands or yolk–sac pattern), (3) SMARCA4 undifferentiated carcinoma, and (4) SMARCA4-deficient subset of teratocarcinosarcoma [51].

### Sinonasal Nonkeratinizing Squamous Cell Carcinoma (SNKSCC) NOS

A morphologically distinct sinonasal carcinoma (prior terminology as transitional, cylindrical cell, Schneiderian, and Ringertz carcinoma) is composed of cytologically atypical neoplastic cells arranged in ribbons that lack maturation and significant keratinization. Two subtypes have been added to the 5th edition WHO Classification of Head and Neck Tumours [52]: (i) HPV-associated – NKSCC defined by the presence of transcriptionally active HPV high risk and (ii) the emerging entity of *DEK::AFF2* NKSCC characterized by recurrent *DEK::AFF2* fusions [53–55]. Morphological differences between NKSCC-NOS and these subtypes of carcinomas are not appreciated.

**Fig. 5:**
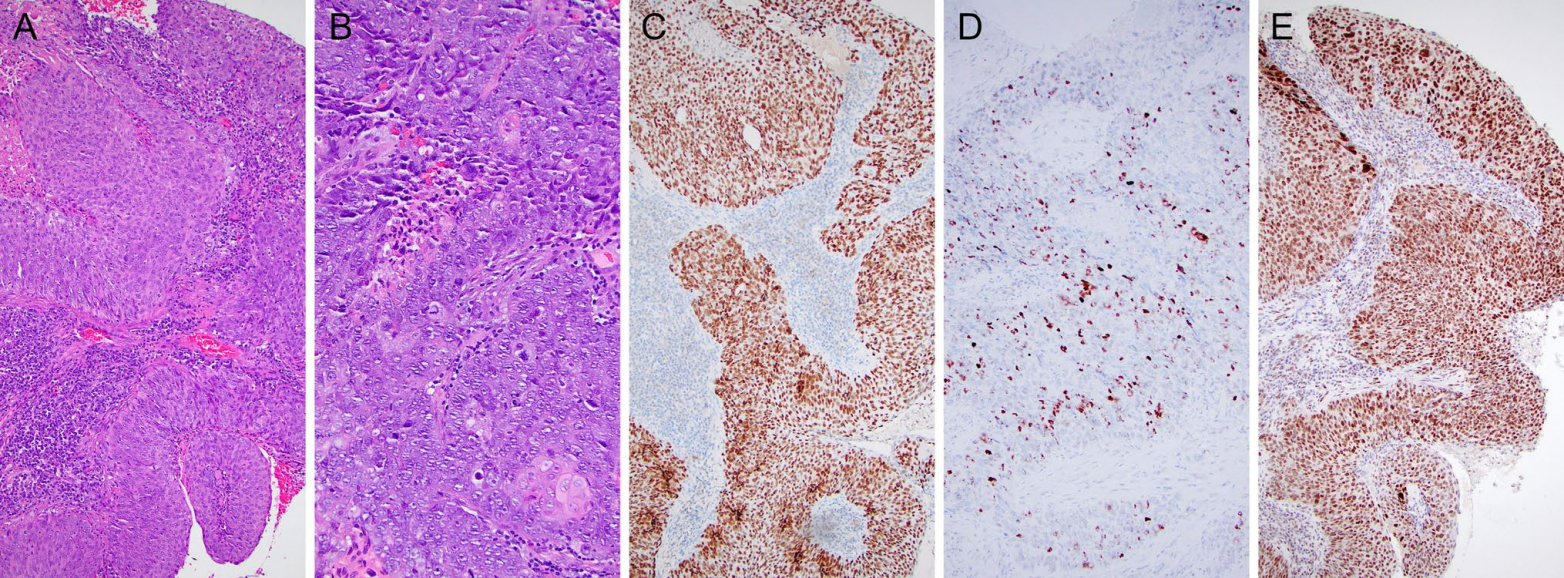
Sinonasal nonkeratinizing SCC, NOS. (**A**, **B**). H&E Nonkeratinizing squamous cell carcinoma with ribboned arrangement (**A**) and discrete keratinization (**B**- 200 × magnification). (**C**) Diffuse positivity with anti-p40 antibody and (**D**) scattered anti-INSM1 reactivity. (**E**) PRAME is diffusely expressed within tumor cells. Viral studies (EBER, HPV-hr) are negative (not illustrated)

SNKSCCs are diffusely positive for keratins CK5/6 and 34ß12 (CK903) and for p63 and p40; negative for synaptophysin, chromogranin, and INSM1, although occasional discrete or focal positivity for neuroendocrine markers is accepted; negative for NUT and EBV; and show retained SMARC expression. An example of NKSCC-NOS is shown in Fig. 5. Methodologies for *DEK::AFF2* fusion confirmation include RNA sequencing, *DEK* FISH, or surrogate AFF2 antibody [56].

### Sinonasal Teratocarcinosarcoma and Sinonasal High-Grade Poorly Differentiated Sinonasal Carcinomas NOS

#### Sinonasal Teratocarcinosarcoma (STCS)

TCS is a rare skull base and sinonasal tract malignant tumor composed of carcinomatous, sarcomatous, and immature neural elements [57]. The most frequent sites are the ethmoid and maxillary sinuses and the nasal cavity in elderly male patients [58].

Morphologically, the tumor is characteristically composed of a high-grade carcinomatous component admixed with sarcomatous and immature neural elements. This tumor causes a diagnostic dilemma if only a dominant component is present on small biopsy samples. The TCS phenotype mirrors its constituent components: cytokeratin immunoreactivity within the epithelial component, CK5/6, p40, p63 for squamous elements, conventional neuroendocrine markers (chromogranin, synaptophysin, INSM1) highlighting the neuroepithelial component (and occasionally focally positive in epithelial), reactivity for myogenic markers (desmin, MyoD1, myogenin), SATB2, and SOX9 in sarcoma elements. Markers of germ cell derivation AFP, PLAP, and hCG are usually negative; however, positivity for SALL4 can be observed, and SALL4 immunohistochemistry appears to be relatively sensitive and specific for the diagnosis of TCS [59, 60]. Recurrent *SMARCA4* alterations resulting in loss of SMARCA4 (BRG1) have been documented in up to 70% of studied TCS cases [61, 62]**.** Aberrant nuclear ß-catenin localization has been reported in a subset of TCS [61].

**Fig. 6:**
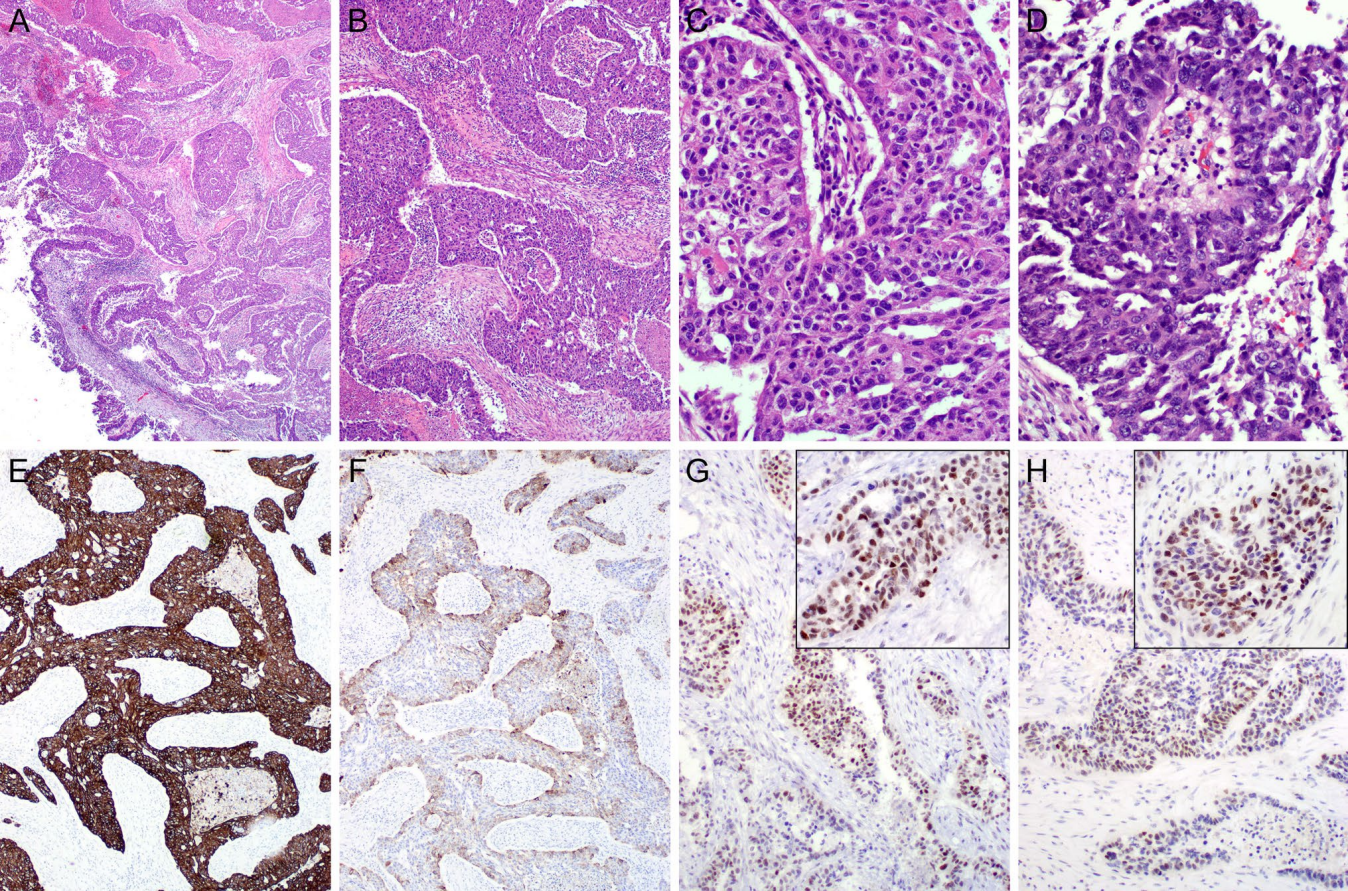
High-grade sinonasal (“Schneiderian”) carcinoma NOS (arising from the middle turbinate). (**A**–**D**) H&Es. The histological appearance is dominated by the presence of surface epithelial dysplastic transformation with endophytic epithelial growth of complex architectural patterns (ribboned, glandular, sieve-like spaces, ciliated neoplastic epithelium, and Schiller-Duval-like elements (**D**) Diffuse immunoreactivity with anti-CK7 (**E**) and limited CK5/6 expression (**F**). No sarcoma elements are identified upon thorough sampling and examination of surgical specimens. The lack of all traditional neuroendocrine and conventional germ cells (AFP, PLAP, glypican, hCG) adds to the diagnostic challenges. SALL4 (**G**) and PRAME (**H**) expressions inform the pluripotential embryonic stem/germ cell origin. Comprehensive molecular NGS studies have not resulted in a more definitive diagnosis

Despite major technological advances instrumental in refining the classification of sinonasal carcinomas, high-grade poorly differentiated sinonasal carcinoma NOS constitutes a temporary default diagnosis for a subset of cases. Figure 6 offers an illustrative example of a high-grade carcinoma arising from the middle turbinate in a middle-aged man (case from author files). The histological appearance is dominated by the presence of surface epithelial dysplastic transformation with endophytic epithelial growth of complex architectural patterns (ribboned, glandular, sieve-like spaces, ciliated neoplastic epithelium, and Schiller-Duval-like elements). No sarcoma elements are identified upon thorough sampling and examination of surgical specimens. The lack of all traditional neuroendocrine and conventional germ cells (AFP, PLAP, glypican, hCG) adds to the diagnostic challenges. SALL4 and PRAME expressions inform the pluripotential embryonic stem/germ cell origin. Comprehensive molecular NGS studies have not resulted in a more definitive diagnosis.

#### Sinonasal Neuroendocrine Carcinoma (SNEC)

Sinonasal neuroendocrine carcinomas (divided into small- and large-cell carcinomas) are almost always high-grade tumors with morphological and immunohistochemical evidence of neuroendocrine differentiation, characterized by a dismal prognosis and a high tendency to produce systemic metastasis. The most common location is the nasal cavity (40%), followed by the ethmoid sinus and maxillary sinuses (~ 20%), sphenoid sinus (13%), and frontal sinus (2%).

The 5th edition WHO Classification of Head and Neck Tumours relocates sinonasal small-cell neuroendocrine carcinomas [63] (SmCNECs) and large-cell neuroendocrine carcinomas [64] (LCNECs) into a dedicated neuroendocrine tumor section in an effort to unify neuroendocrine tumor terminology across organ systems. Regardless, their diagnostic criteria have not changed, with a minimum of 10 mitoses per 2 mm^2^ and Ki67 > 20% being mandatory.

The immunohistochemical profile includes positivity for cytokeratins AE1/AE3, Cam5.2, CK8/18 (frequently with a perinuclear dot distribution), and neuroendocrine markers (synaptophysin, chromogranin, INSM1- variable expression). The Ki67 mitotic index is more than 20%, usually ~ 70–80%). SNECs may benefit from induction chemotherapy followed by concurrent chemoradiation; surgery can be performed in nonresponsive cases or as a salvage treatment.

There is an ongoing effort to apply and validate novel lung small-cell (SCLC) molecular subtyping and biomarker-driven therapy for SNEC. According to RNA expression with validation at the protein levels of the transcription factors *ASCL1*, *NEUROD1*, *POU2F3,* and *YAP1*, four SCLC subtypes have emerged: SCLC-A (ASCL1-driven), SCLC-N (NEUROD1-driven), SCLC-P (ASCL1/NEUROD1-double negative with POU2F3 expression), and SCLC-Y (YAP1-related and NOS) and SCLC-I (inflamed gene signature), which share the last subtype [65–69]. SCLC-Is exhibit the greatest response to the addition of immunotherapy to chemotherapy, while the other subtypes each have distinct vulnerabilities, including to inhibitors of PARP, Aurora kinases, or BCL-2 [68].

**Fig. 7:**
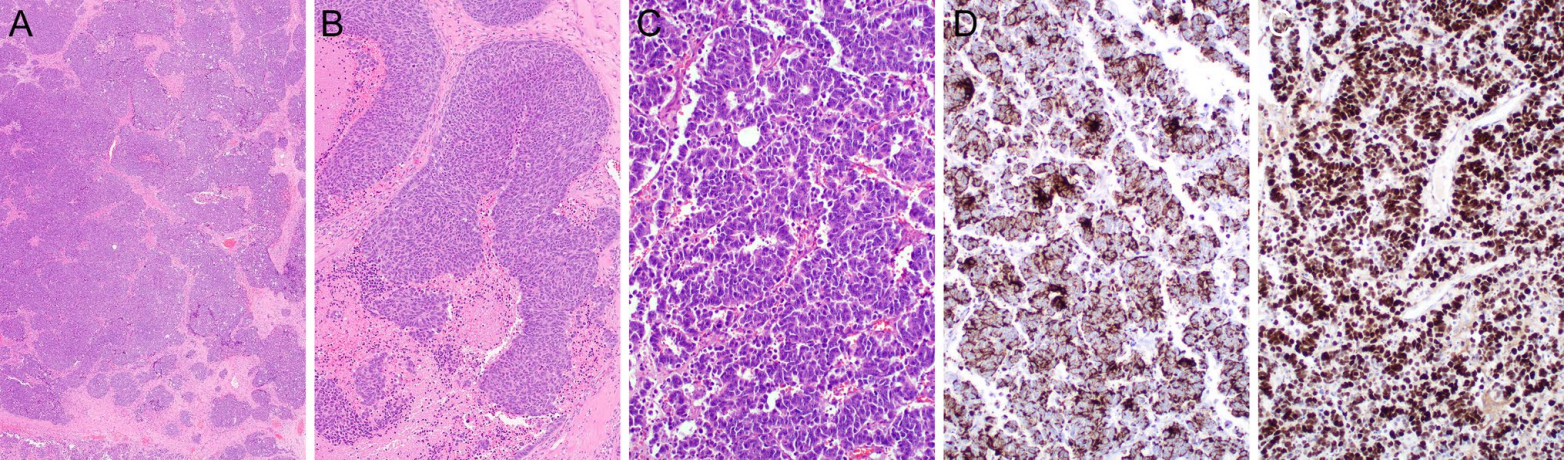
Sinonasal large-cell neuroendocrine carcinoma (LCNEC). (**A**–**C**) H&Es. Classical morphology- large cell size (> 3 lymphocytes), granular, stippled chromatin; architecture- organoid, peripheral palisading, trabeculae, pseudorosettes; large confluent central necrosis, mitoses (typically > 10 MF/2 mm^2^) (**D**). Diffuse immunoreactivity with anti-Cam5.2 (including dot pattern) and (**E**) diffuse ASCL1 expression

Achaete-scute homolog 1 (*ASCL1*) is a powerful player in modulating neuroendocrine differentiation in tumor cells. ASH1 expression levels are inversely associated with the degree of tumor differentiation (high-grade tumors show increased expression of this protein), which correlates well with studies indicating that the expression of ASCL1 appears to be restricted to immature cells [70–73]. ASCL1 expression has been documented previously in high-grade carcinomas of the sinonasal tract [32, 74]. Figure 7 depicts a nasal cavity SLCNEC with classical morphology and diffuse ASCL1 expression.

Neurogenic differentiation factor 1 (NEUROD1)-high SCLC is associated with higher overall neuroendocrine marker expression, equivalent to that of ASCL1-dominant tumors [65, 67, 68].

POU class 2 homeobox 3 (POU2F3) is a marker of chemosensory tuft cells (brush cells in the lung airways), and its expression is associated with low expression levels of neuroendocrine markers in lung SCLC. Yes-associated protein 1 (YAP1), a transcription regulator in the HIPPO growth signaling pathway, was found to be expressed in a subset of nonneuroendocrine SCLCs [65, 67, 68]. Koh et al. recently showed that molecular classification of SCLCs can be applied to extrapulmonary neuroendocrine carcinomas/poorly differentiated carcinomas (EP-NEC/PDCs) and that POU2F3-dominant or YAP1-dominant subtypes are distinct subtypes of EP-NEC/PDCs [75].

## Neuroectodermal Neoplasms

### Olfactory Neuroblastoma (ONB) and Olfactory Carcinoma

ONB represents the “sine-qua-non” for SRBCTs, arranged in submucosal lobules with a neurofibrillary background set in a richly vascular or hyalinized stroma. Immunohistochemical profiles including a spectrum of different cell lineages are crucial to the diagnosis. ONB is usually diffusely positive for chromogranin and synaptophysin, with S100 protein-positive sustentacular cells characteristically highlighting the periphery of tumor lobules. Sustentacular cells tend to be attenuated/disappear with histological grading progression. Negative staining includes muscle markers, leukocyte common antigen CD45 (LCA), CD99, and p40. Up to one-third of ONBs will exhibit focal staining for cytokeratin (most common are low molecular keratins Cam 5.2 and CK 8/18). ONB can express somatostatin receptors (SSTR2 in particular); somatostatin analogs can be used for diagnosis, especially in cases of metastatic disease [76, 77]. Recently, Zunitch et. Al, utilized an integrated human-mouse single cell atlas of the nasal mucosa, including the olfactory epithelium, to identify transcriptomic programs that link ONB to a specific population of stem,/progenitor cells – olfactory globose basal cells (GBCs) [78]. The authors further advocate that expression of a GBC transcription factor (NEUROD1) distinguishes both low-and high-grade ONB from SNUC. Furthermore, their study identified a reproducible subpopulation of highly proliferative ONB cells expressing the GBC stemness marker EZH2, suggesting that EZH2 inhibition may play a role in the targeted treatment of ONB [78].

ONB exhibits epithelial differentiation to varying degrees, which is a unique feature that sets it apart from peripheral neuroblastoma in other regions; melanocytic, myogenic, and neural differentiation has been occasionally reported [79]. These are manifested in the forms of glands, squamous morules, and rhabdomyoblastic or ganglioneuroblastic differentiation. Divergence may be encountered in pretreatment or posttreatment samples and can change after treatment; such divergence should be accepted only when a pathognomonic feature of ONB is identified (neurofibrillary stroma or sustentacular cells) or in a recurrence/posttreatment resection of an otherwise typical ONB [46].

**Fig. 8:**
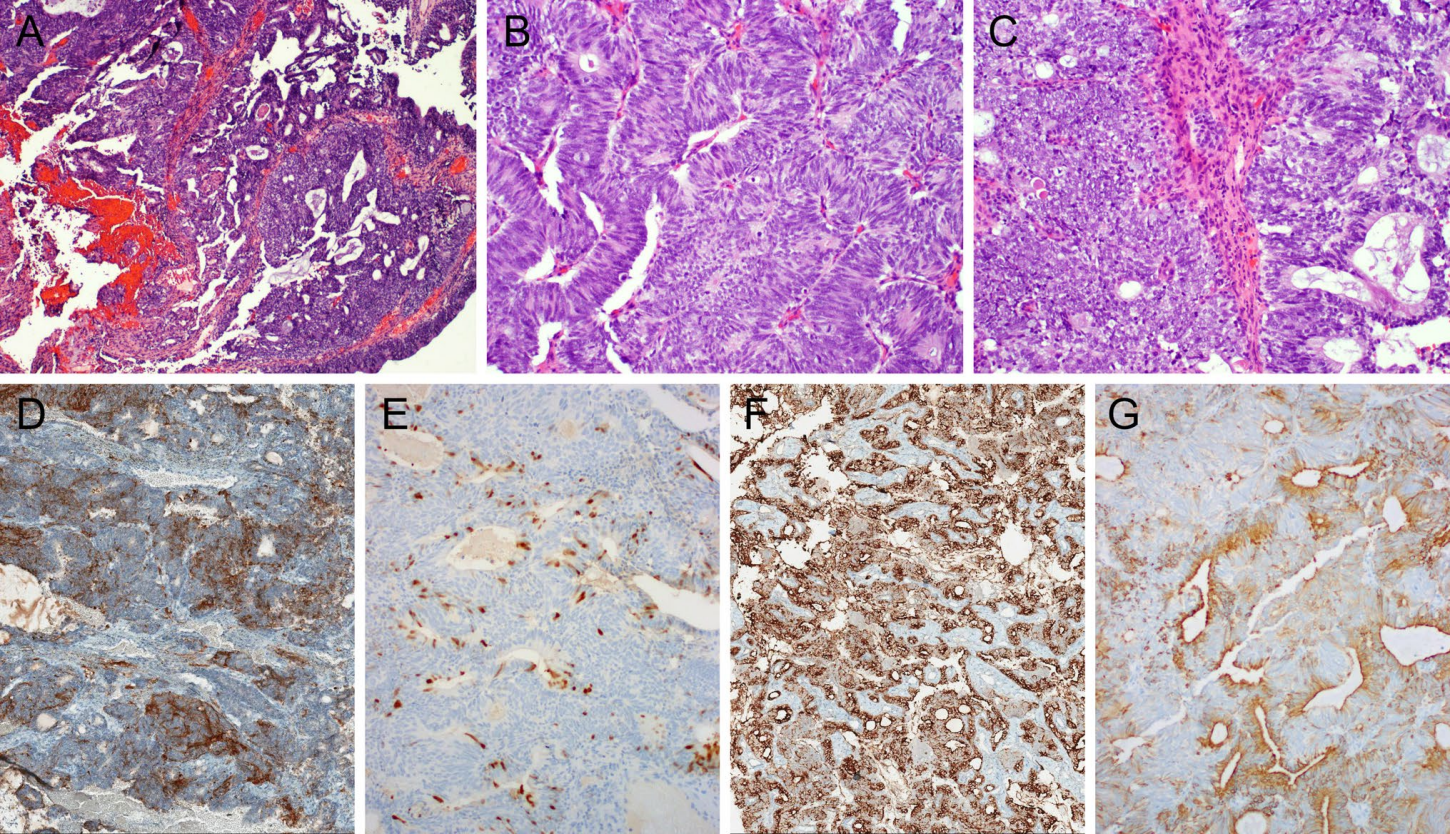
Olfactory carcinoma. Morpho-phenotypical findings included (i) lobulated and solid growth; (ii) rosettes and/or neurofibrillary stroma; (iii) high-grade cytology; (iv) glands, frequently ciliated (**A**–**C** H&Es); and (v) extensive keratin and neuroendocrine immunoreactivity, variable sustentacular S100 component. Immunoperoxidase staining with anti-synaptophysin (**D**), anti-S100, anti-Cam5.2 (**F**), anti-AE1/AE3 (**G**)

Rooper et al. reviewed a total of 53 sinonasal tumors with neuroepithelial differentiation [79]. The majority of these arose in the superior nasal cavity and high stage (Kadish-Morita) at presentation. Morpho-phenotypical findings included (i) lobulated and solid growth; (ii) rosettes and/or neurofibrillary stroma; (iii) high-grade cytology; (iv) glands, frequently ciliated; and (v) extensive keratin and neuroendocrine immunoreactivity, variable sustentacular S100 component. The authors advocated for a unifying nomenclature as re-instauration of the olfactory carcinoma taxonomy, with olfactory carcinoma being defined by high-grade keratin-positive neuroectodermal cells with frequent intermixed glands and recurrent *Wnt* pathway, *ARID1A*, and *RUNX1* alteration [80, 81]. Figure 8 illustrates olfactory carcinoma.

### Malignant Mucosal Melanoma (MM)

Sinonasal MM is the most aggressive sinonasal tumor and is currently characterized by early recurrence and high dissemination rates regardless of the treatment adopted. Free-margin surgery is the mainstay of treatment since it is generally considered a radio-resistant cancer. Cells are histologically comparable to melanoma arising in other locations and may be plasmacytoid, epithelioid, spindle, or rhabdoid. Architectural patterns are varied and nonspecific, and pigmentation is variable. When lacking melanin pigment, immunohistochemistry becomes paramount: S100 protein, SOX10, and PRAME are usually strongly positive, while other melanocytic markers (HMB45, tyrosinase, melan A, and MITF) have variable expression. The well-known mutated genes involved in cutaneous melanoma have only a marginal role in MM, and the infrequent rate of BRAF V600E mutation observed in MM limits the efficacy of BRAF inhibitors. Recently, immunotherapy has shown promising results in selected cases, both in neoadjuvant and adjuvant settings, especially in terms of decreased systemic spread of disease [82].

### Ectopic Pituitary Adenoma

Pituitary adenomas mainly occur in the sphenoid bone and sinuses either as a separate lesion or as an extension from a primary adenoma arising in the sella; embryonic residue along the Rathke pouch formation is the presumed derivation [84]. Approximately half of patients manifest hormonal abnormalities. Histologically, an ectopic pituitary adenoma is identical to a conventional pituitary adenoma with monotonous round cells.

**Fig. 9:**
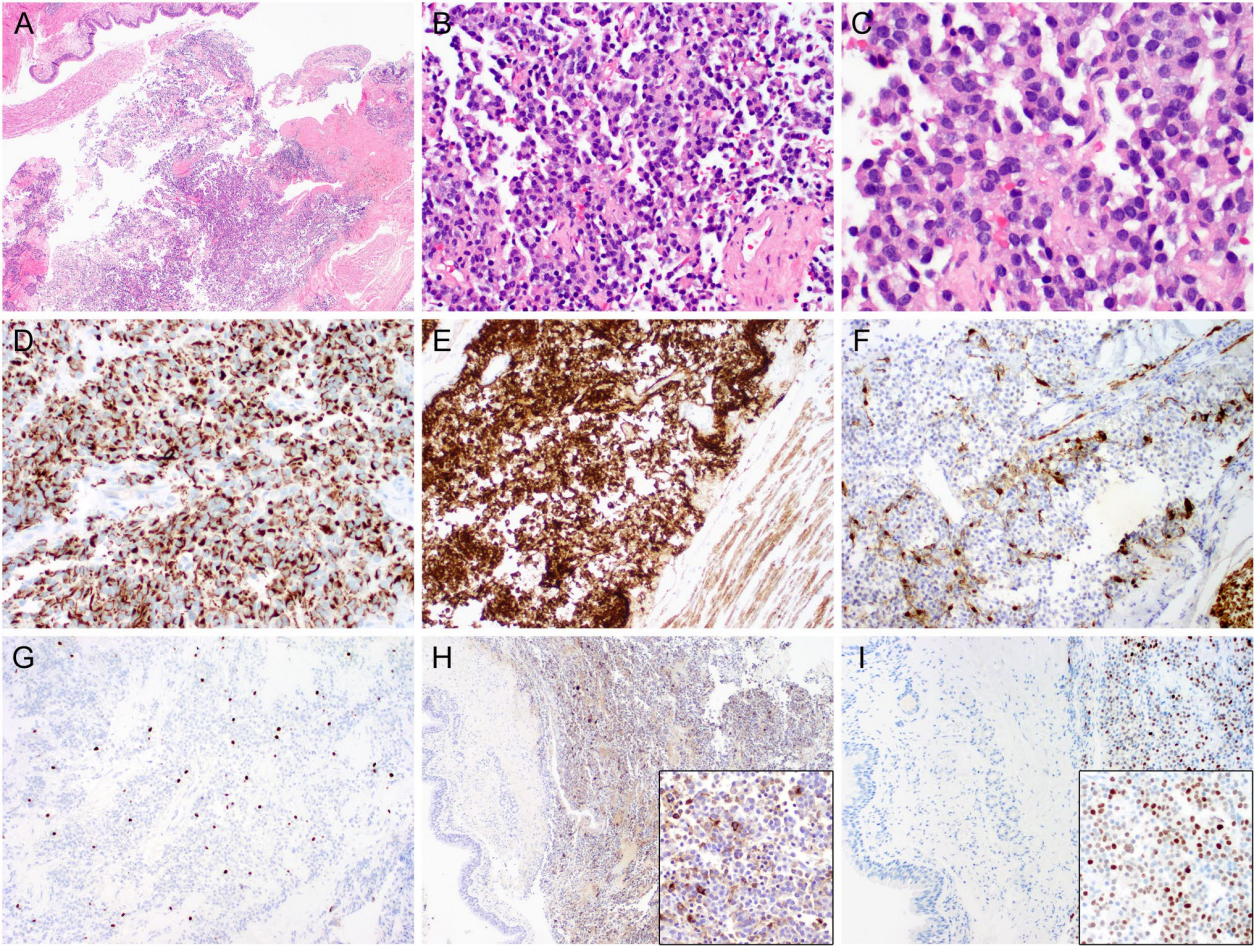
Ectopic pituitary adenoma. (**A**–**C**) H&Es. Histologically, an ectopic pituitary adenoma is identical to a conventional pituitary adenoma with monotonous round cells. This neoplasm should be differentiated from carcinoid tumors, olfactory neuroblastoma, and other small undifferentiated tumors at these locations, as the expression of Cam5.2 is a common pitfall (**D**); diffuse positivity for synaptophysin (**E**) with folliculostellate (FSC) cells highlighted by S100 (**F**); proliferation rate Ki67 is low, 2% (**G**). Immunohistochemical staining for hormonal receptors, especially for ACTH (**H**) and pituitary transcription factor Pit1 (**I**), is helpful

This neoplasm should be differentiated from carcinoid tumors, olfactory neuroblastoma, and other small undifferentiated tumors at these locations, as the expression of Cam5.2 is a common pitfall. Immunohistochemical staining for hormonal receptors, especially for ACTH and prolactin, and pituitary transcription factors Pit1 and T-pit is very helpful (Fig. 9).

### Ewing Family Tumors (EFTs)

Ewing family tumors (previously known as Ewing sarcoma (EWS) and primitive neuroectodermal tumor (PNET)) are interrelated primitive round cell malignancies of neuroectodermal derivation. They represent a spectrum of morphologic entities that share common molecular genetic features, e.g., fusions involving members of the *FET* and *ETS* gene families. They are uncommon childhood and young adult tumors affecting the skull base and sinonasal tract regions in approximately 5% of patients. The maxillary sinus and the nasal fossa are commonly affected sites. Histologically, the tumor presents in sheets and nests of densely uniform small-cell proliferation, with occasional rosette formation. CD99 (MIC2) is diffusely positive (membranous pattern); nuclear expression of Fli-1 and NKX2.2 also supports the diagnosis; chromogranin, synaptophysin, or low-molecular-weight cytokeratin is expressed in a subset of EFTs. The adamantinoma-like variant is strongly positive for high-molecular-weight cytokeratin, p40 and p63. NGS (with *EWSR1* or *FUS* rearrangement) and FISH for EWSR1 are confirmatory for diagnosis (supplemental Fig. 1).

## Other SRCTs

### Rhabdomyosarcoma (RMS)

This is a relatively uncommon mesenchymal malignancy of the skull base region. Rhabdomyosarcoma is the most common sarcoma of the head and neck and is the most frequent childhood sarcoma. The sinonasal tract and the nasopharynx are the most affected sites. The embryonal type (ERMS) is the most common type in children, while the alveolar type (ARMS) predominates in an older age group.

Immunohistochemical markers, including desmin, myo-D, and myogenin, are necessary for the diagnosis, especially of the embryonal form. Most alveolar subtype RMSs harbor a *PAX3* or *PAX7::FOXO1* fusion. A PAX3 variant translocation that partners with *NCOA* family members in place of *FOXO1* has been described in RMS [83].

### Hematolymphoid Malignancies

#### Plasmablastic Lymphoma (PBL)

Plasmablastic lymphoma is a high-grade B-cell lymphoma with a plasmacytoid appearance and plasma cell phenotype and is frequently associated with MYC overexpression. The sinonasal tract may be affected, as this lymphoma occurs predominantly at extranodal sites. The tumor cells express plasma cell markers (CD38, MUM1, CD138) and light chain restriction (either kappa or lambda); B-cell markers (CD20, PAX5) are negative; and EBER is positive (at least in 50% of cases) (supplemental Fig. 2).

#### Extramedullary Plasmacytoma

A similar morphology and phenotype make extramedullary plasmacytoma challenging to distinguish from PBL. Extramedullary plasmacytoma is defined by the absence of bone marrow involvement and end organ damage, which are found in PBL. Diagnostic confirmation can be achieved by immunohistochemistry or in situ hybridization for immunoglobulin mRNA with identification of light chain restriction. The majority of plasmacytomas are negative for EBER and have no MYC rearrangements. Where distinction between PBL and plasmacytoma is not possible, a descriptive diagnosis of plasmablastic neoplasm is suggested (supplemental Fig. 2).

## Conclusions

The diagnostic challenges of sinonasal SRBCTs have been addressed to date by introducing newly described entities, reclassifying conventional lesions, and redesigning treatment modalities for these rare tumors after conducting multi-institutional cohort retrospective analysis. The accuracy of diagnosis is sine-qua-non for the therapeutic approach and prognosis of patients with sinonasal cancer.

**Fig. 10:**
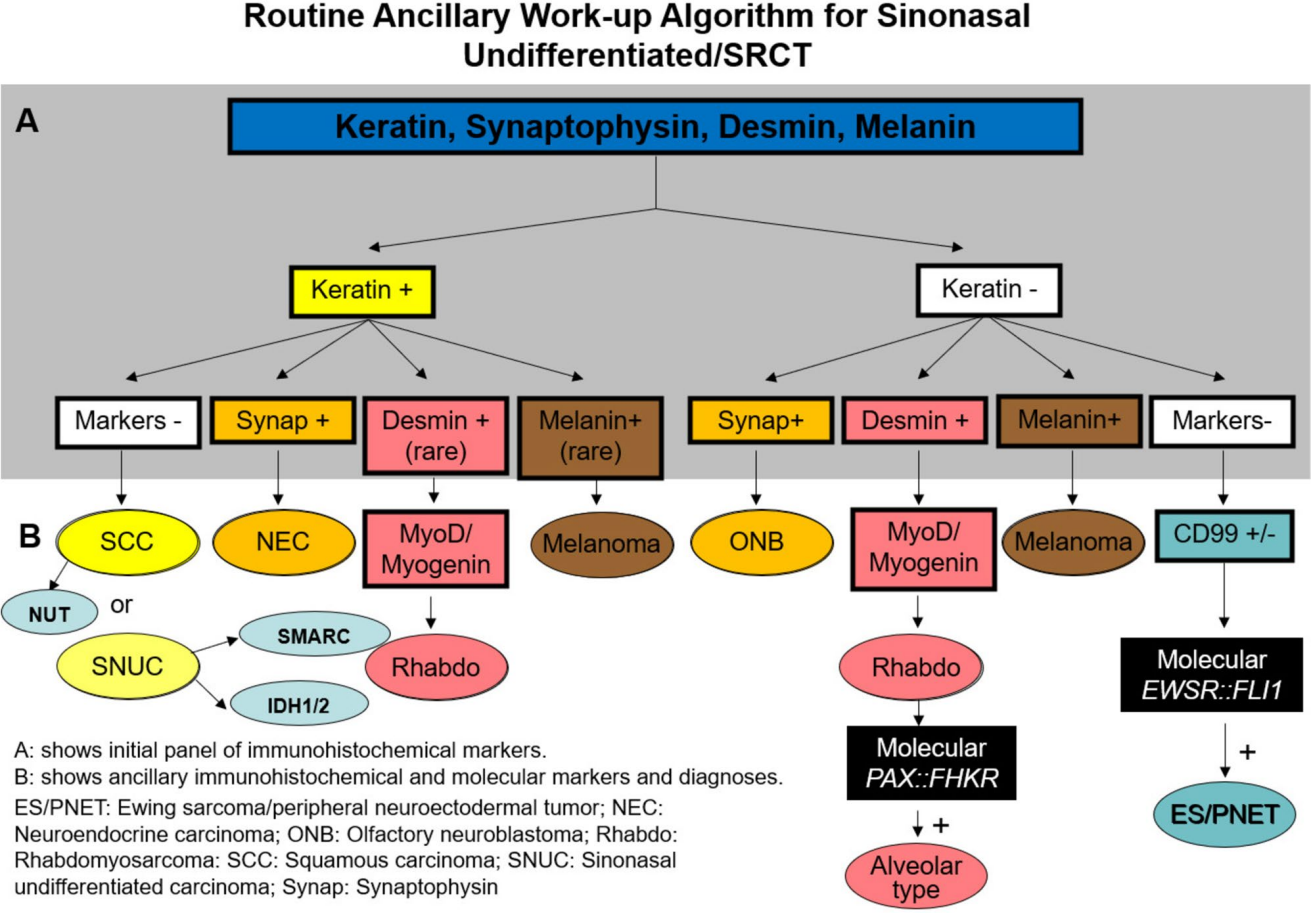
Flowchart of routine ancillary work-up algorithm for sinonasal undifferentiated/SRBCT

The initial goal is to establish the lineage and triage the tissue for ancillary and molecular studies. Ideally, differentiation is reached based on multiple factors, including more than one stain. If the result is equivocal, rather than interpreting as “weak positive/essentially negative, additional confirmatory studies should be performed. Awareness of “aberrant expression” is important to avoid confusion. Many diagnostic errors occur because too few markers are assessed. Figure 10 illustrates a routine ancillary work-up algorithm for sinonasal undifferentiated/SRBCT.

The request for specific molecular testing is based on differential diagnostic considerations and implies a continuous screening of literature and updates. The lack of access for pathologists worldwide to the latest technologies and the limited nature of these SRBCTs (with an inability to perform extensive exploratory testing) are several drawbacks. Table 1 summarizes a wish list of diagnostic “hacks” and theragnostic ancillary studies. Laforga and Abdullah recently formalized a diagnostic algorithm for these tumors by integrating morphological clues, immunohistochemical markers and morphological investigations [85]. The algorithm is presented through a user-friendly web interface and a mobile phone application, with the intent to help pathologists establish a correct diagnosis of a challenging SRBCT and researchers perform retrospective analysis of archival cases. Prior to this, several algorithms based on another single institutional experience have been proposed to meet the need for integrated diagnosis for skull base malignancies [86, 87].

**Table 1:** Wish list for diagnostic hacks and theragnostic ancillary studies (standalone ability/surrogate, in bold) in sinonasal undifferentiated/SRCT

	Diagnostic hacks		Complementary/Theragnostic
	IHC, ISH	Molecular (FISH, NGS)	
NC	**NUT**	NUTM1 (FISH) *BRD4::NUTM1* *BRD3::NUTM1*	Sox2, PRAME (IHC)
SLEC	**EBER**		SSTR2 (IHC)
SNUC IDH2-mutant	**IDH1/2 (pR132/172)**	***IDH2p.R172***	
SWI/SNF-deficient carcinoma	**SMARCB1(INI1/BAF47)** **SMARCA4 (BRG1)**		
SNKSCC	HPV-high risk (ISH RNAScope)		PRAME (IHC)
	AFF2	***DEK::AFF2***	
TCS	SMARCA4, SALL4, beta-catenin	*CTNNB1*	
High-grade NEC (SmCNEC, LCNEC)	**ASCL1**, NEUROD1		POU2F3, YAP1 (IHC)
ONB	NEUROD1		SSTR2, EZH2 (IHC)
Olfactory carcinoma	ASCL1, NEUROD1	*CTNNB1*, *ARID1A*, *RUNX1*	ASCL1, PRAME (IHC)
MMM	**PRAME**		
EPA (sphenoid sinus)	**Pit1**, **T-Pit**		
EFT	NKX2.2	**EWSR1** (FISH) *EWS::FLI1* and/or other *EWSR1* rearrangements *FUS::ERG* and/or other *FUS* rearrangements	
Rhabdomyosarcoma		**FOXO1** (FISH) *PAX3::FOXO1* *PAX7:FOXO1*	

*NC* NUT carcinoma, *SLEC* sinonasal lymphoepithelial carcinoma, *SNUC* sinonasal undifferentiated carcinoma, *SNKCC* sinonasal nonkeratinizing squamous cell carcinoma, *TCS* teratocarcinosarcoma, *ONB* olfactory neuroblastoma, *High-grade NEc* high-grade neuroendocrine carcinoma, *SmNEC* small-cell neuroendocrine carcinoma, *LCNEC* large-cell neuroendocrine carcinoma, *MMM* malignant mucosal melanoma, *EFT* Ewing family tumors, *EPA* ectopic pituitary adenoma, *RMS* rhabdomyosarcoma

Sinonasal SRCTs are rare and heterogeneous tumors, with an imperative need for novel diagnostic, prognostic, and therapeutic biomarkers. Validation of the current findings and building a comprehensive model of carcinogenesis for each sinonasal tumor require multi-institutional efforts. With more novel targeted therapies being developed, options for personalized treatment of sinonasal cancers are growing, with the goal of improved survival for this challenging group of tumors.

## Supplementary Information

Below is the link to the electronic supplementary material.

Supplementary file1 (JPG 5968 KB)

Supplementary file2 (JPG 6678 KB)

## Data Availability

Not applicable.
