# Author Correction: Top IHC/ISH Hacks for and Molecular Surrogates of Poorly Differentiated Sinonasal Small Round Cell Tumors

**DOI:** 10.1007/s12105-024-01636-3

**Published:** 2024-04-18

**Authors:** Diana Bell

**Affiliations:** Anatomic Pathology, Disease Team Alignment: Head and Neck, City of Hope Comprehensive Cancer Center, 1500 E Duarte Rd, Duarte, CA 91010 USA

**Correction to: Head and Neck Pathology (2024) 18:2** 10.1007/s12105-023-01608-z

The original version of this article unfortunately contained mistakes in the captions of Figs. 1, 2 and 3. The captions for the part figures E, F in Fig. 1, part figure D, E in Fig. 2 and part figure C in Fig. 3 were missing. They read wrongly as:

**Fig. 1 **NUT carcinoma. (**A**, **B**) Salient morphological features–monotonous proliferation of round small-to-medium-sized cells abrupt keratinization and foci of squamous differentiation with larger cells with eosinophilic cytoplasm and pearl formation; (**C**, **D**) Immunoperoxidase staining with anti-SOX2 (**C**) and anti-PRAME (**D**), as promising anticancer targets

**Fig. 2 **Sinonasal lymphoepithelial carcinoma. (**A**) H&E-conventional morphology, with syncytial undifferentiated malignant cells set in a rich lymphoid background (Regaud pattern). (**B**) Presence of Epstein–Barr virus confirmed by EBER in situ hybridization. Immunostainings with (**C**) PRAME and (**D**) SSTR2

**Fig. 3 **Sinonasal IDH2-mutated carcinoma. (**A**) H&E-Submucosal lobules of undifferentiated malignant cells, with large nuclei and prominent nucleoli (**B**) H&E. Diffuse immunoreactivity with anti-mutant IDH1/2 pR132/172, confirmed by molecular NGS

They should have read as follows:

**Fig. 1 **NUT carcinoma. (**A**, **B**) Salient morphological features–monotonous proliferation of round small-to-medium-sized cells; abrupt keratinization and foci of squamous differentiation with larger cells with eosinophilic cytoplasm and pearl formation (**C**) Immunoperoxidase staining with (**D**) anti-NUT, (**E**) anti-SOX2, (**F**) anti-PRAME

**Fig. 2 **Sinonasal lymphoepithelial carcinoma. (**A, B**) H&E, (**C**) Presence of Epstein–Barr virus confirmed by EBER in situ hybridization. Immunostaining with (**D**) PRAME and (**E**) SSTR2

**Fig. 3 **Sinonasal IDH2-mutated carcinoma. (**A**) H&E-Submucosal lobules of undifferentiated malignant cells, with large nuclei and prominent nucleoli (**B**) H&E. Diffuse immunoreactivity with anti-mutant IDH1/2 pR132/172 (**C**)

The original article has been corrected.

