# Supplementary figures and images for: Top IHC/ISH Hacks for and Molecular Surrogates of Poorly Differentiated Sinonasal Small Round Cell Tumors
Source: Head Neck Pathol. 2024 Feb 5;18(1):2. doi: 10.1007/s12105-023-01608-z (PMC10844182; doi:10.1007/s12105-023-01608-z)

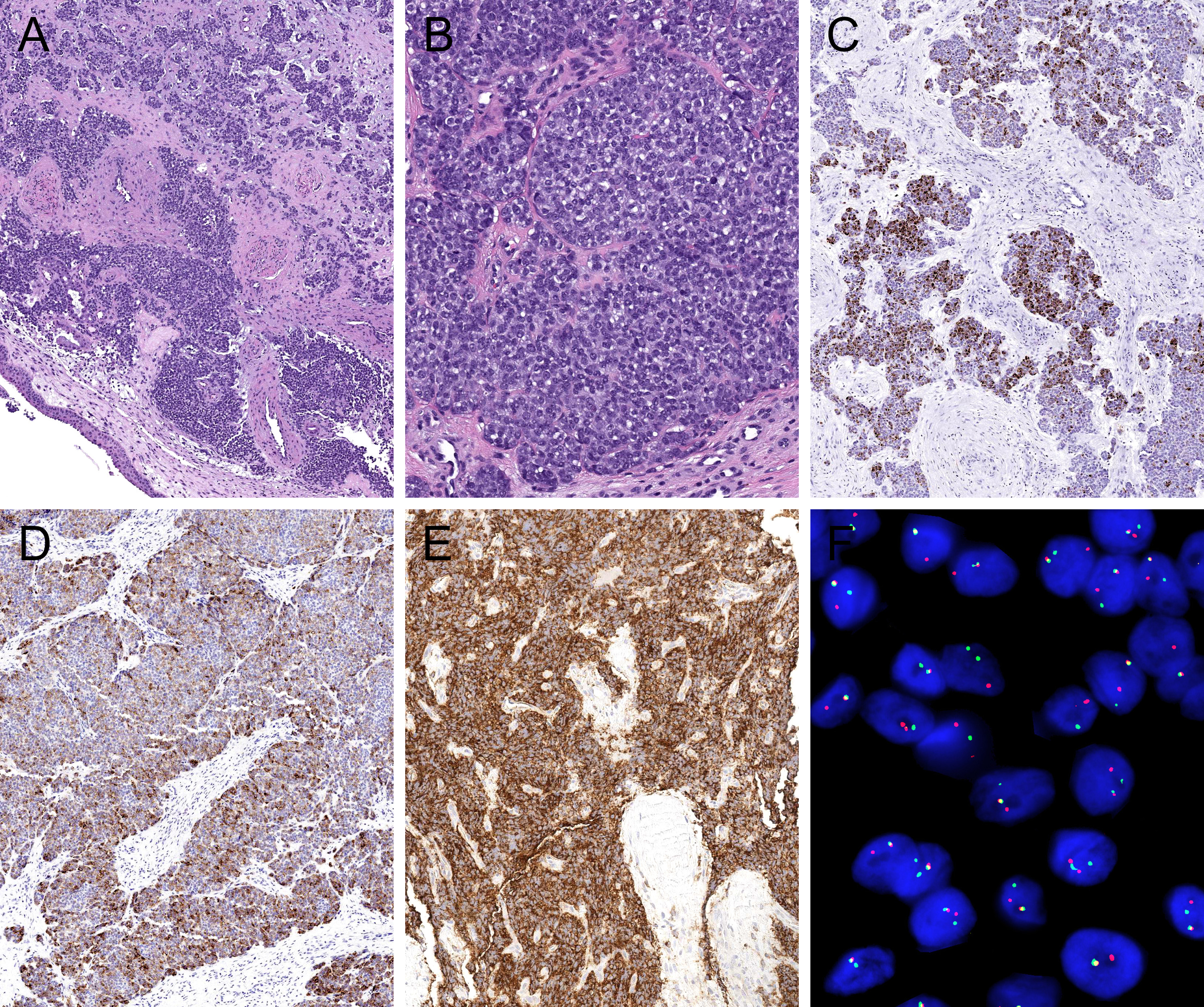

Supplement: Supplementary file 1 — Supplementary file1 (JPG 5968 KB) [file 12105_2023_1608_MOESM1_ESM.jpg]

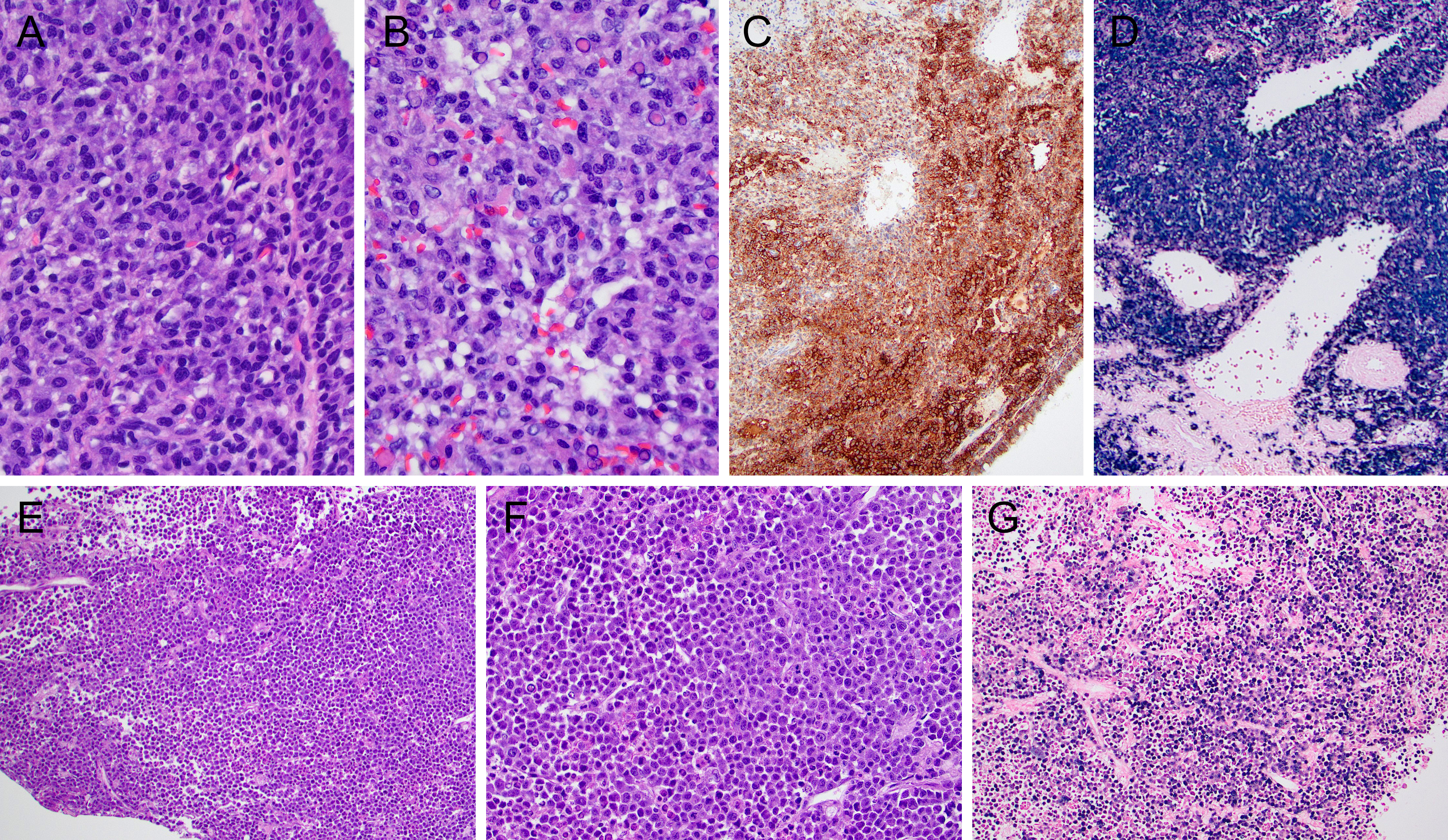

Supplement: Supplementary file 2 — Supplementary file2 (JPG 6678 KB) [file 12105_2023_1608_MOESM2_ESM.jpg]
